# Osteoporosis as Perceived by Saudi Physicians: A Cross-Sectional Study of Quality of Practice and Current Barriers in Management

**DOI:** 10.7759/cureus.49578

**Published:** 2023-11-28

**Authors:** Rana A Alahmadi, Hind M Aljabri, Nuha M Alharbi, Dalia M Alghamdi, Sadeen T Shahbar, Yousef Al-Saleh, Salwa Alaidarous

**Affiliations:** 1 Medicine, King Saud Bin Abdulaziz University for Health Sciences College of Medicine, Jeddah, SAU; 2 Medicine, National Guard Hospital, Jeddah, SAU; 3 Medicine, King Abdulaziz Medical City, Riyadh, SAU; 4 Endocrinology and Metabolism, King Saud Bin Abdulaziz University for Health Sciences, Riyadh, SAU; 5 Endocrinology, King Abdulaziz Medical City, King Saud Bin Abdulaziz University for Health Sciences, Ministry of National Guards Health Affairs, Jeddah, SAU

**Keywords:** physicians' practice, knowledge, saudi, frax, osteoporosis

## Abstract

Background

Osteoporosis (OP) is a state of abnormal bone quality and architecture that leads to fragility fractures, with lifetime costs reaching 16.27 billion Saudi Arabian Riyal (SAR).

Methods

An electronic survey was distributed to physicians from July 2020 to May 2021 to assess the quality of the practice of physicians toward OP and barriers in OP management in Saudi Arabia. Specialties included were endocrinology, general medicine, family medicine, primary care, orthopedic surgery, rheumatology, obstetrics and gynecology, and geriatrics.

Results

A total of 177 surveys were eligible (55.9% female and 44.1% male). The majority were family consultants (42.9%). In terms of knowledge, 18.1% of our sample recognized all risk factors, and 24.9% recognized all indications to assess bone density. A central dual-energy X-ray absorptiometry (DEXA) was accessible to only 49.4% of the sample. Over 80% of the sample performed comprehensive laboratory workup. Although 68.4% of participants were aware of fracture risk assessment (FRAX), 53.7% used it in their practice. The most cited barrier was a lack of physicians' awareness (80.2%), followed by a lack of patients' awareness (63.6%). The specialty was significantly associated with the awareness of the densitometry certificate (P-value < 0.0001) and the use of FRAX (P-value = 0.0001).

Conclusion

Our results revealed a below-satisfactory quality of practice among Saudi physicians toward OP. Additionally, our results identified many gaps in knowledge and many barriers to optimal care.

## Introduction

Osteoporosis (OP) is a condition characterized by low bone density and abnormal quality and architecture that predisposes bones to fractures under little stress. These fractures are described as fragility fractures and are usually the only symptom of OP [1]. In a 2021 meta-analysis of 86 studies on the prevalence of OP, Salari et al. [2] uncovered a worldwide prevalence of 23.1% among females and 11.7% among males. An epidemiological study including Saudi nationals aged between 50 and 79 years showed that 34% of healthy females and 30.7% of healthy males were osteoporotic [3]. To illustrate the economic burden of the disease, Sadat-Ali et al. [4] estimated the lifetime costs of OP-related femoral fractures in the Eastern Province alone to be 564.75 million Saudi Arabian Riyal (SAR).

Studies on Saudi physicians' approach to OP have been limited in number and generally show a mixed image of the lack of knowledge, insufficient clinical practice, and multiple other barriers to providing appropriate care [5-7]. Our literature review has highlighted the scarcity of studies evaluating the quality of the healthcare delivered to OP patients in Saudi Arabia, and most were conducted on a city level only.

This study aimed to assess the quality of the practice of physicians in Saudi Arabia regarding their approach to the diagnosis and management of OP and to investigate the current barriers to providing optimal OP management. Special emphasis was placed on investigating the physicians' use of dual-energy X-ray absorptiometry (DEXA), which is the gold standard in the diagnosis of OP [8]. Finally, we explored the physicians' familiarity with the fracture risk assessment (FRAX) tool, which estimates the 10-year probability of a major osteoporotic fracture in susceptible individuals [9].

## Materials and methods

A cross-sectional study was performed using a web-based questionnaire distributed electronically to consultant physicians in Saudi Arabia. The study was conducted between July 2020 and May 2021. The respondents were reached via social media, phone numbers, and medical societies of interest.

Consultants of endocrinology, general medicine, family medicine, primary care, orthopedic surgery, rheumatology, obstetrics and gynecology, and geriatrics were included, based on their potential involvement in the management and treatment of OP. All consultants were required to be practicing in Saudi Arabia. A convenient sampling approach was applied since the official registry of all physicians in the country in different specialties was not available. As such, no prior sample size calculation was implemented.

Data collection tool

The instrument used for data collection is the questionnaire attached in the Appendices. The original questionnaire was obtained from Beshyah et al.'s research on physicians' perceptions and practices in the management of OP in the Middle East and North Africa [10] and was modified (Appendices). The questionnaire was validated through content validity by two endocrinology consultants and a biostatistician. The questionnaire included 36 questions on demographic information as explanatory variables, the quality of practice as outcome variables, and barriers to management as possible contributing factors. The details of the three questionnaire domains can be found in the Appendices (S1). The questionnaire accommodated different types of practice, and data collectors were recruited to expand the reach of the questionnaire beyond the authors' city of residence.

Data analysis

Descriptive analysis was presented using frequency and percentages. Chi-square tests were used to evaluate the association between the demographic variables (namely, specialty and years of practice) and responses regarding the quality of practice. Fisher's exact test was used when needed. A P-value of <0.05 was considered statistically significant. Statistical analyses were performed using JMP version 8.1 (SAS Institute Inc., Cary, NC) and Statistical Package for Social Sciences (SPSS) version 21 (IBM SPSS Statistics, Armonk, NY) for Windows. Missing responses to some of the questions were reported without imputation. All supporting information can be downloaded at https://rb.gy/jxbaqc.

Ethical consideration

The study was approved by the Institutional Review Board of King Abdullah International Medical Research Center (protocol number: JED-20-427780-53416). Information on study objectives and consent were displayed on the first page of every electronic questionnaire (Appendices). This study was conducted in compliance with the ethical principles of the Declaration of Helsinki.

## Results

Physicians' characteristics

A total of 177 questionnaires that met the inclusion criteria were received online from July 2020 to May 2021. Over half (55.9%) of our respondents were female physicians. Over two-thirds of the respondents (76.8%) had a governmental type of practice, and over half (52.5%) had over 15 years of practice. The majority of respondents were family physicians (42.9%), followed by endocrinologists (23.7%). Most (80.2%) physicians reported treating osteoporotic patients, of whom 95 (53.7%) managed fewer than 10 patients per month. The majority (58.8%) of encountered patients were already diagnosed with OP, while 48.6% were newly screened patients (Table 1).

**Table 1:** Demographic characteristics of the participants (N = 177) OP, osteoporosis; DEXA, dual-energy X-ray absorptiometry

Characteristics		n (%)
City of practice		
Jeddah	94 (53.1)	
Riyadh	48 (27.1)	
Others		35 (19.8)
Gender		
Female		99 (55.9)
Male		78 (44.1)
Specialty		
Endocrinology		42 (23.7)
General medicine		6 (3.4)
Family medicine		76 (42.9)
General practitioner (primary care)		3 (1.7)
Orthopedic surgery		11 (6.2)
Rheumatology		15 (8.5)
Obstetrics and gynecology		19 (10.7)
Geriatrics		1 (0.6)
Others		4 (2.3)
Professional grade		
Consultant		176 (99.4)
Others: general practitioner		1 (0.6)
Years of practice		
≤15 years		84 (47.5)
>15 years		93 (52.5)
Type of clinical practice (checklist)		
Governmental		136 (76.8)
Research and teaching		42 (23.7)
Ministry of Health		45 (25.4)
Primary healthcare		34 (19.2)
Others		12 (6.8)
Encounter and treat OP patients		
Yes		142 (80.2)
No		35 (19.8)
Number of OP patients currently managed per month		
<10		95 (53.7)
≥10		48 (27.1)
Not applicable		34 (19.2)
Typical OP patient encountered (checklist)		
Referred from primary care for DEXA screening		53 (29.9)
Already diagnosed with osteopenia or osteoporosis		104 (58.8)
New fragility fracture		58 (32.8)
Newly screened patient at your clinic		86 (48.6)
Not applicable		30 (16.9)

Knowledge

Overall knowledge of the risk factors for OP was inadequate. In total, less than a fifth (18.1%) of the sample had complete knowledge of the risk factors (evaluated as correctly recognized 10 out of the 10 risk factors in the questionnaire), while the remaining responses were distributed across scores of 4-9 (Table 2). Regarding bone mineral density (BMD) assessment, complete knowledge of its seven indications was demonstrated by only a quarter (24.9%) of our respondents. Most of the remaining respondents scored between 4 and 6, corresponding to 53% of responses (Appendices, S3). A minority of physicians (19.2%) reported consistency in assessing patients' BMD, while 33.9% only did so sometimes (Appendices, S4). A central DEXA was only accessible to half (49.4%) of the respondents, and 6.2% had no accessible densitometry device. The rates of accessibility to other devices can be found in Appendices (S5).

**Table 2:** Risk factors to OP as perceived by the physicians ^a^Any level of activity below the minimum of walking 150 minutes per week ^b^Completing one year since the last menstrual cycle ^c^Additional risk factors added by participants including medications, BMI, and vitamin D deficiency OP: osteoporosis

Risk factors	Score		n (%)
Smoking	10 out 10		32 (18.1)
Poor exercise^a^	9 out 10		28 (15.8)
Elderly	8 out 10		26 (14.7)
Post-menopause^b^	7 out 10		19 (10.7)
Poor nutrition	6 out 10		19 (10.7)
Steroid use	5 out 10		19 (10.7)
Family history	4 out 10		17 (9.6)
Rheumatoid arthritis or any other connective tissue disease	3 out 10		6 (3.4)
Diabetes mellitus	2 out 10		5 (2.8)
Previous fragility fractures	1 out 10		6 (3.4)
Others^c^			

The Appendices (S6) shows the distribution of awareness on multiple OP indicators/signs. In our questionnaire, 17.5% were certified densitometrists (Table 3). Almost a third (29.9%) of the respondents made sure to always review and confirm BMD findings (Appendices, S4), while the remaining 70.1% relied on radiologists or automatically generated reports to varying extents (Table 3).

**Table 3:** Densitometry certification and reporting

Answers	n (%)
Awareness of the densitometry certificate	
Yes	90 (50.8)
No	87 (49.2)
Received a densitometry certificate	
Yes	31 (17.5)
No	146 (82.5)
Reporter of the bone densitometry in practice	
A radiologist with a densitometry certificate	41 (23.2)
A radiologist without a densitometry certificate	15 (8.5)
Automatically generated reports	12 (6.8)
Combination of radiologist and automatically generated reports	34 (19.2)
Not sure	75 (42.4)

Regarding further workup following BMD assessment, most (85.3%) participants obtained blood tests for assessing bone health before initiating OP treatment. Screening tests for both vitamin D and calcium sufficiency in a patient with fragility fractures were carried out by 87.6% of the physicians (Appendices, S8).

FRAX

**Figure 1:**
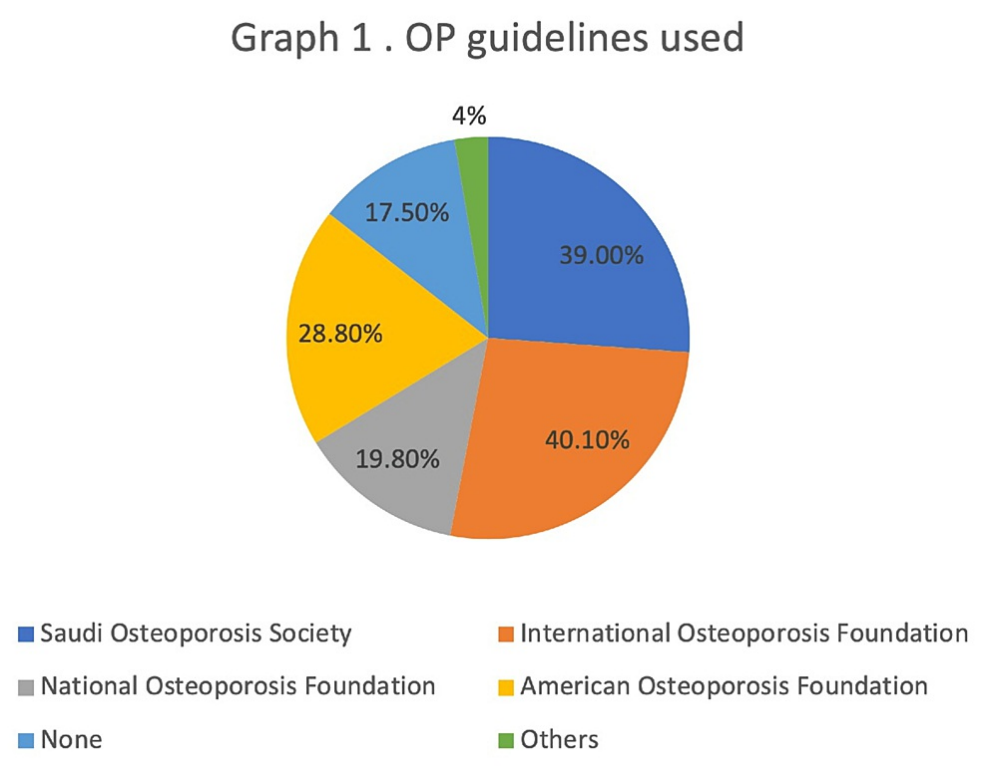
OP guidelines used OP: osteoporosis

Over half (68.4%) of the respondents were aware of FRAX as a tool for fracture risk assessment. However, only 53.7% of them used it in their practice, and the US model was the most used (23.2%) (Table 4). Most respondents (71.8%) used both risk factors and BMD, without FRAX, to assess for the risk of fracture in their patients (Appendices, S10). As for the guidelines, the International Osteoporosis Foundation guideline was the most cited (40.1%) for the diagnosis and treatment of OP (Figure 1).

**Table 4:** Use of FRAX in practice FRAX: fracture risk assessment

Answers	n (%)
Awareness of FRAX	
Yes	121 (68.4)
No	56 (31.6)
Frequency of using FRAX	
Always	32 (18.1)
Frequently	41 (23.2)
Sometimes	36 (20.3)
Rarely	25 (14.1)
Never	43 (24.3)
Country model used (checklist)	
USA	41 (23.2)
Abu Dhabi	24 (13.6)
Jordan	23 (13)
Kuwait	21 (11.9)
Lebanon	20 (11.3)
United Kingdom	4 (2.3)
Palestine	2 (1.1)
Morocco	1 (0.6)
Tunisia	1 (0.6)
Others	15 (8.5)
Not applicable	65 (36.7)

As for the physicians' practice regarding bisphosphonate prescription, an individualized treatment approach was the most favored for both oral (58.8%) and intravenous (IV, 70.1%) preparations (Appendices, S12).

Barriers

In the initial screening of OP, the cost was the most commonly reported reason for not obtaining blood tests before OP treatment, reported by 7.9% of the respondents. The reasons for not using FRAX were the following: not knowing how to use the tool (15.3%), a lack of a model for the country of practice (8.5%), being time-consuming (6.2%), the inapplicability of the tool (3.4%), and no internet access in the clinic (3.4%).

In terms of the prescription of anti-osteoporotic medications, 92% of our respondents were eligible to prescribe oral bisphosphonates, denosumab (41.4%), IV bisphosphonates (37%), teriparatide (28.4%), and selective estrogen receptor modulators (SERMs, 24.7%). Oral bisphosphonates were the most available (83.3%), followed by denosumab (51.2%), IV bisphosphonates (43.2%), teriparatide (41.4%), and SERMs (21%). None were available for 14.2% of our respondents.

Physicians were asked to choose their greatest barriers in delivering OP care. Most cited the lack of physician (80.2%) and patient awareness (63.6%), followed by costs of medication (30.9%), concerns about the safety of the medication (27.8%), restrictions by health insurance or managed care (24.7%), the lack of time (22.8%), and concerns about the medications' effectiveness (16%).

Physicians were asked which specialties they thought must shoulder the responsibilities of screening and managing OP. For screening, the majority (84.6%) pointed to primary care/family medicine, while the second majority (66.7%) believed it was the endocrinologists' responsibility. As for medical management, endocrinology was mentioned most frequently (86.4%), followed by primary care/family medicine (63%) (Table 5). The prevalence of each barrier for every question can be found in the Appendices (S13).

**Table 5:** Barriers to optimum OP management *162/177 participants who answered this question OP, osteoporosis; FRAX, fracture risk assessment

Answers	n (%)
Common reasons for not obtaining blood tests before initiating treatment in patients (checklist)	
Tests are irrelevant	10 (5.6)
Cost	14 (7.9)
Patient's refusal	4 (2.3)
Difficulties in interpreting the results	4 (2.3)
Lack of time	8 (4.5)
Not sure which tests are most essential to order	10 (5.6)
Others	8 (4.5)
Main reason for not using FRAX in practice	
There is no model for my country	15 (8.5)
FRAX is time-consuming	11 (6.2)
FRAX tool is not applicable to my practice	6 (3.4)
I do not know how to use it	27 (15.3)
No internet access in my clinic	6 (3.4)
OP treatment medications*	
Privileged/can prescribe	
Oral bisphosphonates	149 (92)
Intravenous bisphosphonates	60 (37)
Selective estrogen receptor modulators (SERMs)	40 (24.7)
Teriparatide	46 (28.4)
Denosumab	67 (41.4)
Available at institution	
Oral bisphosphonates	135 (83.3)
Intravenous bisphosphonates	70 (43.2)
Selective estrogen receptor modulators (SERMs)	34 (21)
Teriparatide	67 (41.4)
Denosumab	83 (51.2)
None	23 (14.2)
Biggest barriers to OP care in practice/country (checklist)*	
Costs of medication	50 (30.9)
Lack of physicians' awareness	130 (80.2)
Lack of patients' awareness	103 (63.6)
Concerns about the safety of medications	45 (27.8)
Restrictions by health insurance or managed care	40 (24.7)
Concerns about the medications' effectiveness	26 (16)
Lack of time	37 (22.8)
Others	7 (4.3)
Specialties whose responsibility is the screening for OP (checklist)*	
Endocrinology	108 (66.7)
General medicine	89 (54.9)
Obstetrics and gynecology	49 (30.2)
Orthopedic surgery	68 (42)
Rheumatology	79 (48.8)
Primary care/family medicine	137 (84.6)
Geriatrics	82 (50.6)
Others	5 (3.1)
Specialties whose responsibility is the medical management of OP (checklist)*	
Endocrinology	140 (86.4)
General medicine	76 (46.9)
Obstetrics and gynecology	23 (14.2)
Orthopedic surgery	50 (30.9)
Rheumatology	82 (50.6)
Primary care/family medicine	102 (63)
Geriatrics	80 (49.4)
Others	4 (2.5)

Associations

There was a significant association between the specialty and the awareness of the densitometry certificate, where 83.3% of those in the specialty of endocrinology were aware of the certification compared to 38.16% in the specialty of family medicine (P-value < 0.001). However, such an association was absent between the specialty and attainment of the certificate (P-value = 0.14). There was a significant association between the specialties of the respondents and their use of FRAX (P-value < 0.001) (Appendices, S14).

No significant association was found between years of practice and knowing the risk factors for OP (P-value = 0.31), knowing the indicators of OP (P-value = 0.49), knowing the indications for a BMD assessment (P-value = 0.40), the frequency of obtaining blood tests (P-value = 0.85), obtaining vitamin D and calcium status (P-value = 0.95), assessing BMD (P-value = 0.40), or reviewing and confirming BMD findings (P-value = 0.10). No significant association was found between the specialty and practice in prescribing oral (P-value = 0.35) or IV bisphosphonates (P-value = 0.05) (Appendices, S14).

## Discussion

Our data suggested that Saudi consultants largely adhered to guidelines and were comprehensive in their laboratory orders for OP. Over half of the surveyed physicians reported using FRAX in their fracture risk assessment. Most physicians also preferred an individualized approach to prescribing anti-osteoporotic agents. Nevertheless, our results showed unsatisfactory levels of knowledge on risk factors and indicators of OP, as well as on indications for a BMD assessment. We have also identified multiple barriers to ideal practice.

In a qualitative study, Alghamdi and Mohammed [5] reported more reassuring results in a survey of 141 Saudi physicians and nurses, where 90.1% were reported to have good knowledge of OP. Although no significant difference was found between the subgroups, the high prevalence could be explained by the inclusion of nurses, who were the majority of respondents and also the group with the higher scores. The results of the study by Al-Musa et al. [6] showed a lower prevalence of appropriate knowledge; among 66 primary healthcare (PHC) physicians, 54.5% had good and 25.8% had poor overall knowledge of OP, while none (0%) had excellent knowledge. In another survey of 540 Iranian family physicians, Mahdaviazad et al. [11] noted a percentage of as low as 14% in knowledge of OP, and this was congruent with our findings. Such predominantly inadequate knowledge is not surprising considering physicians' self-reported lack of training and preparedness as noted in the studies of Al-Musa et al. [6] and Mahdaviazad et al. [11], as well as international studies [12]. Additionally, a Riyadh-based survey of 364 PHC physicians by Saeedi et al. [7] revealed that only 3.9% had a specialized OP program at their institution.

For diagnosis, Ha et al. [13] reported that only 59% of 100 surveyed Korean physicians tested routinely for vitamin D insufficiency. Here, we reported this to be >80%, which may be explained by the frequent use of one or more of the guidelines listed in the questionnaire, as only 17.5% admitted to not following one. In addition, our questionnaire showed significantly higher awareness of national guidelines (57.1%), compared to those reported by Al-Musa et al. [6] (18.2%) and Mahdaviazad et al. [11] (23.5%). This could also explain the better laboratory practices among our respondents compared to others.

Al Saleh et al. [14] previously reported that only 15.5% of their 264 hip fracture patients were assessed for BMD, and <20% had bone-related laboratory investigations done. Similarly, our questionnaire showed that a minority of physicians assessed for BMD (19.2% always and 33.3% frequently), although this should be interpreted in light of central DEXA being available to only 49.4% and peripheral DEXA to 10.5% of physicians.

The use of FRAX was not possible for 18.7% of our respondents who admitted to not knowing how to use the tool or had no internet access in the clinic, a finding similar to that reported by Ha et al. [13] (12.71%). Fifteen (8.5%) of our respondents thought that the lack of a national model was a significant setback, one that was finally overcome with the announcement of the Saudi model of FRAX in November 2021 [15].

The last objective of this study was to determine the barriers physicians face. Starting with screening for OP, similar to our results, Ha et al. [13] found cost to be the most reported barrier in obtaining blood tests. However, this was reported by 35% of their sample as opposed to 7.9% of our sample, and this may be due to differences in healthcare systems. Accessible densitometry devices were less frequently reported here compared to international studies [10,13], highlighting an important area for improvement in screening approaches.

Here, oral bisphosphonates were not available to 16.7% of the respondents, while 14.2% had none of the listed medications. These numbers were more reassuring than those reported by Al-Musa et al. [6] in 2013, where 75.8% of physicians marked the unavailability of medications as a barrier. Nonetheless, our rates remained higher than those reported by Beshyah et al. [10], who found that only 3.8% of their surveyed physicians in the Middle East and North Africa had no access to oral bisphosphonates. This and previous studies have also reported that medication-related concerns pose as barriers. Kim et al. [16] previously interviewed 100 doctors, the majority of whom expressed concerns about medication effectiveness (64%) and safety (66.3%), which far exceed our results (16% for effectiveness and 27.8% for safety), a difference that may be due to the authors' focus on severe rather than all types of OP cases.

Most of our respondents (80.2%) thought that the lack of physicians' awareness was the greatest barrier. However, a study by Ha et al. [13] found the lack of patients' awareness to be the greatest barrier (reported by 50% of physicians). This was also reported by Beshyah et al. [10], although the difference between the two barriers in their study was negligible. Such findings emphasize the need for education on both sides of the patient-doctor encounter in the context of OP.

A limitation of this study was the sample size of 177, which was not enough to safely generalize findings to all Saudi physicians. Additionally, our sample was overly representative of the western region of the country. We attempted to overcome these limitations by extending the survey period and using online reminders when possible. We also hired data collectors from other cities with greater access to the online healthcare community in their respective cities to limit selection bias. Physical reminders would have been more efficient, increasing the cooperation of doctors, who tend to display low response rates [17], but they were not feasible during the COVID-19 pandemic.

This was a novel study aiming to explain the approach of consultants of multiple specialties and on a national level when it comes to the diagnosis and management of OP. We recommend future research dedicated to investigating possible interventions to raise awareness of the disease in the community at large, as well as a more specific investigation of the barriers physicians face in their path to alleviating the burden of OP.

## Conclusions

The results of this questionnaire revealed a below-satisfactory practice quality in the management of OP among Saudi physicians. Additionally, there were several gaps identified in the knowledge of risk factors associated with OP and the indications for a BMD assessment. This study also highlighted several barriers that Saudi physicians encounter in managing OP. These findings may help guide plans to optimize OP care in Saudi Arabia. Potential improvement plans may include providing professional education to physicians, improving patients' awareness of the disease, and reformulating a current and population-oriented guideline for OP diagnosis and management.

## Appendices

Questionnaire

Demographic Features

1. Mark your city of practice:

○ Riyadh

○ Jeddah

○ Madinah

○ Dammam

○ Others: _____________

2. Name your hospital of practice:

Answer:

3. Please indicate your gender:

○ Male

○ Female

4. Please indicate your specialty:

○ Endocrinology

○ General medicine

○ Obstetrics and gynecology

○ Orthopedic surgery

○ Rheumatology

○ Family medicine

○ General practitioner (primary care)

○ Geriatrics

○ Others: _____________

5. Please indicate your current professional grade:

○ Consultant

○ Specialist/fellow

○ Resident

○ Others: _____________

6. Please indicate your years of practice:

○ Less than three years

○ 3-5 years

○ 6 -10 years

○ 11-15 years

○ More than 15 years

7. Please indicate the type of your clinical practice: (Mark all that apply)

○ Governmental

○ Research and teaching

○ Ministry of Health

○ Primary healthcare

○ Others: _____________

8. Do you encounter and treat patients with osteoporosis?

○ Yes

○ No

9. How many patients with osteoporosis do you currently manage per month?

○ <10

○ 10-20

○ 21-50

○ 51-100

○ >100

○ Not applicable

10. Who is the typical osteoporotic patient you encounter? (Mark all that apply)

○ Referred from primary care for DEXA screening

○ Already diagnosed with osteopenia or osteoporosis

○ New fragility fracture

○ Newly screened patient at your clinic

○ Not applicable

Quality of Practice

11. Are you aware of the Saudi guidelines for osteoporosis?

○ Yes

○ No

○ Not sure

12. What guideline do you use? (Mark all that apply)

○ Saudi Osteoporosis Society

○ International Osteoporosis Foundation

○ National Osteoporosis Foundation

○ American Association of Clinical Endocrinologists

○ None

○ Others:___________________

13. How often do you assess bone density in your patients?

○ Always

○ Frequently

○ Sometimes

○ Rarely

○ Never

14. What do you believe are indications for bone density assessment for osteopenia/osteoporosis? (Mark all that apply)

○ All females ≥40 years who have sustained low-trauma fragility fracture

○ All females >60 years of age

○ Previous fragility fracture or maternal history of hip fracture

○ Premature menopause (age < 40)

○ Prolonged secondary amenorrhea (>1 year)

○ Patients who had X-ray findings suggestive of osteoporosis (fragility fracture, loss of height, or thoracic kyphosis) or are on drugs associated with bone loss

○ Adults with primary hyperparathyroidism or other diseases or nutritional conditions associated with bone loss

15. Are you aware of the densitometry certification?

○ Yes

○ No

16. Have you ever received a densitometry certificate?

○ Yes

○ No

17. Who reports the bone densitometry in your practice?

○ A radiologist with a densitometry certificate

○ A radiologist without a densitometry certificate

○ Automatically generated reports

○ Combination of radiologist and automatically generated reports

○ Not sure

18. How often do you review the scan/printout and confirm the findings?

○ Always

○ Frequently

○ Sometimes

○ Rarely

○ Never

19. Which of the following risk factors do you regularly investigate in patients you suspect with osteoporosis? (Mark all that apply)

○ Smoking

○ Poor exercise

○ Elderly

○ Post-menopause

○ Poor nutrition

○ Steroid use

○ Family history

○ Rheumatoid arthritis or any other connective tissue disease

○ Diabetes mellitus

○ Previous fragility fractures

○ Others:______________

20. Which of the following may indicate osteoporosis in a patient? (Mark all that apply)

○ Kyphosis

○ Loss of height

○ Lumbar lordosis

○ Fractures

○ Back pain

○ Not applicable

21. Do you routinely obtain blood tests for the assessment of bone health before initiating treatment with anti-osteoporotic agents in your patients?

○ Yes

○ No

22. Do you screen for vitamin D and calcium sufficiency status in your patients with osteoporosis and fragility fractures?

○ No

○ Only vitamin D

○ Only calcium

○ Yes, both

23. If you do not obtain blood tests before initiating treatment with anti-osteoporotic agents in your patients, what is the reason? (Mark all that apply)

○ I think that the tests are irrelevant

○ Cost

○ Patient's refusal

○ Difficulties in interpreting the results

○ Lack of time

○ Not sure which tests are most essential to order

○ Others: _____________

24. What is your practice in regard to prescribing oral bisphosphonates? (Mark all that apply)

○ Re-evaluation for the need for the continued use of oral bisphosphonates after 3-5 years

○ Change to an alternate medication after five years

○ Complete discontinuation of treatment after five years

○ Drug holiday and re-evaluation

○ Individualized treatment depending on the case

25. What is your practice in regard to prescribing IV bisphosphonates? (Mark all that apply)

○ Re-evaluation for the need for the continued use of oral bisphosphonates after 2-3 years

○ Change to an alternate medication after five years

○ Complete discontinuation of treatment after five years

○ Drug holiday and re-evaluation

○ Individualized treatment depending on the case

26. Are you aware of FRAX?

○ Yes

○ No

27. What tools do you use in your practice for fracture risk assessment? (Mark all that apply)

○ Risk factors alone

○ BMD alone

○ Risk factors and BMD

○ FRAX

28. How often do you use FRAX?

○ Always

○ Frequently

○ Sometimes

○ Rarely

○ Never

29. If you use FRAX in your practice, which country model do you use? (Mark all that apply)

○ Jordan

○ Lebanon

○ Morocco

○ Palestine

○ Tunisia

○ USA

○ United Kingdom

○ Others: _____________

○ Not applicable

30. If you do not use FRAX in your practice, what is the main reason?

○ There is no model for my country

○ FRAX is time-consuming

○ No internet access in my clinic

○ FRAX tool is not applicable to my practice

○ I do not know how to use it

○ Others: _____________

Barriers

31. What kind of densitometry device do you have access to in your clinical practice?

○ Central DEXA

○ Peripheral DEXA

○ Central quantitative computed tomography (QCT)

○ No accessible device

○ Not sure

32. What medications are available in your institution for the treatment of osteoporosis? (Mark all that apply)

○ Oral bisphosphonates

○ Intravenous bisphosphonates

○ Selective estrogen receptor modulators (SERMs)

○ Teriparatide

○ Denosumab

○ None

33. Please check all the ones you are privileged/are able to prescribe: (Mark all that apply)

○ Oral bisphosphonates

○ Intravenous bisphosphonates

○ Selective estrogen receptor modulators (SERMs)

○ Teriparatide

○ Denosumab

34. In your opinion, whose responsibility it is to screen for osteoporosis? (Mark all that apply)

○ Endocrinology

○ General medicine

○ Obstetrics and gynecology

○ Orthopedic surgery

○ Rheumatology

○ Primary care/family medicine

○ Geriatrics

○ Others: _____________

35. In your opinion, who is responsible for the medical management of osteoporosis? (Mark all that apply)

○ Endocrinology

○ General medicine

○ Obstetrics and gynecology

○ Orthopedic surgery

○ Rheumatology

○ Primary care/family medicine

○ Geriatrics

○ Others: _____________

36. What do you perceive as the biggest barrier to osteoporosis care in your practice/country? (Mark all that apply)

○ Costs of medication

○ Lack of physicians' awareness

○ Lack of patients' awareness

○ Concerns about the safety of medications

○ Restrictions by health insurance or managed care

○ Concerns about the medications' effectiveness

○ Lack of time

○ Others: _____________

Supplementary materials

The following supporting information can be downloaded at https://rb.gy/jxbaqc: Table S1: The three domains of the survey and their corresponding questions; Table S2: Risk factors to OP as perceived by the physicians; Table S3: Known indications for BMD assessment; Table S4: BMD assessment practice; Table S5: Accessible densitometry devices in practice; Table S6: Indicators of OP in a patient; Table S7: Densitometry certification and reporting; Table S8: Laboratory workup; Table S9: Use of FRAX in practice; Table S10: Assessment tools used for fracture risk; Table S11: OP guidelines; Table S12: Bisphosphonate prescription practices; Table S13: Barriers in practice; Table S14: Associations between demographics and aspects of practice; Table S15: Years of practice by knowledge of risk factors, indicators of OP, and BMD indications.

## References

[1] Sözen T, Özışık L, Başaran NÇ (2017). An overview and management of osteoporosis. Eur J Rheumatol.

[2] Salari N, Ghasemi H, Mohammadi L, Behzadi MH, Rabieenia E, Shohaimi S, Mohammadi M (2021). The global prevalence of osteoporosis in the world: a comprehensive systematic review and meta-analysis. J Orthop Surg Res.

[3] Alwahhabi BK (2015). Osteoporosis in Saudi Arabia. Are we doing enough?. Saudi Med J.

[4] Sadat-Ali M, Al-Dakheel DA, Azam MQ (2015). Reassessment of osteoporosis-related femoral fractures and economic burden in Saudi Arabia. Arch Osteoporos.

[5] Alghamdi MA, Mohammed AG (2018). Knowledge and awareness of osteoporosis among Saudi physicians and nurses: a cross-sectional study. Open Access Maced J Med Sci.

[6] Al-Musa HM, Alassmi M, AlMoria A, Alghamdi H, Alfaifi S (2013). Knowledge, practice and barriers in management of osteoporosis. Biomed Res.

[7] Saeedi MY, Al-Amri F, Mohamed A, Ibrahim AK (2014). Knowledge, attitude and practice towards osteoporosis among primary health care physicians in Riyadh, Saudi Arabia. J Public Heal.

[8] Dimai HP (2017). Use of dual-energy X-ray absorptiometry (DXA) for diagnosis and fracture risk assessment; WHO-criteria, T- and Z-score, and reference databases. Bone.

[9] Kanis JA, Harvey NC, Johansson H, Odén A, Leslie WD, McCloskey EV (2017). FRAX update. J Clin Densitom.

[10] Beshyah SA, Al-Saleh Y, El-Hajj Fuleihan G (2019). Management of osteoporosis in the Middle East and North Africa: a survey of physicians' perceptions and practices. Arch Osteoporos.

[11] Mahdaviazad H, Keshtkar V, Emami MJ (2018). Osteoporosis guideline awareness among Iranian family physicians: results of a knowledge, attitudes, and practices survey. Prim Health Care Res Dev.

[12] Pritchard J, Karampatos S, Ioannidis G (2016). Osteoporosis guideline implementation in family medicine using electronic medical records: survey of learning needs and barriers. Can Fam Physician.

[13] Ha YC, Lee YK, Lim YT, Jang SM, Shin CS (2014). Physicians' attitudes to contemporary issues on osteoporosis management in Korea. J Bone Metab.

[14] Al Saleh Y, El Seid ME, Ruhaiyem ME (2020). Characteristics and outcomes of osteoporotic hip fractures: treatment gaps in a tertiary care center in Riyadh, Saudi Arabia. Aging Clin Exp Res.

[15] Al-Daghri NM, Sabico S, Al-Saleh Y (2021). The application of FRAX in Saudi Arabia. Arch Osteoporos.

[16] Kim JH, Park YS, Oh KJ, Lee SY, Lee SY, Lee SK, Chung YS (2016). Perception of severe osteoporosis amongst medical doctors in South Korea: awareness, impact, and treatment. Osteoporos Sarcopenia.

[17] Cunningham CT, Quan H, Hemmelgarn B (2015). Exploring physician specialist response rates to web-based surveys. BMC Med Res Methodol.

